# Natural sciences at the service of art and cultural heritage: an interdisciplinary area in development and important challenges

**DOI:** 10.1111/1751-7915.13766

**Published:** 2021-02-10

**Authors:** Guadalupe Piñar, Katja Sterflinger

**Affiliations:** ^1^ Institute of Natural Sciences and Technology in the Arts (INTK) Academy of Fine Arts Vienna Vienna Austria

## Abstract

Our cultural heritage is a common asset that tells the story of our shared past, is part of our origin and identity and has wide social relevance. Our works of art and our heritage must be enjoyed, appreciated and preserved for future generations. To this end, a wide and varied group of professionals, including conservators, restorers, curators, bibliographers, historians, archivists, but also scientists, such as biologists, chemists, physicists and bioinformaticians, work side by side to preserve our cultural heritage. Working together in this wide range of disciplines included in the so‐called ‘heritage sciences’ is the only plausible way to contribute to the sustainable preservation of our heritage. The great progress made in recent years in conservation and restoration work, but also in the natural sciences considered within heritage science, has provided powerful tools and strategies for analytical and experimental research into historical and cultural objects that open up new frontiers for their diagnosis, monitoring and protection. Here we highlight some of the advances and challenges faced by the natural sciences at the service of art.

## Graphical abstract

The natural sciences are part of the interdisciplinary field of heritage sciences. Here we show the amazing technological development they have undergone in recent years and the challenges this fascinating field faces.

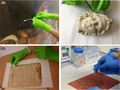

Our Cultural Heritage is a common asset that tells the story of our shared past, is part of our origin and identity and has a wide social relevance. Museums, archives, libraries and heritage sites around the world preserve priceless works of art, which must be enjoyed, appreciated, and preserved for future generations. A wide and varied group of professionals, including conservators, restorers, curators, bibliographers, historians, archivists, but also scientists, such as biologists, chemists, physicists and bioinformaticians, work side by side to preserve our cultural heritage. Working together in this wide range of disciplines included in the so‐called ‘heritage sciences’ is the only plausible way to contribute to the sustainable preservation of our patrimony. Nevertheless, heritage science is a field that has been scientifically recognised as such not so long ago, perhaps the reason for this lies precisely in its interdisciplinary nature.

The great progress made in recent years in conservation and restoration work, but also in the natural sciences considered within the heritage sciences, has provided powerful tools and strategies for analytical and experimental research into historical and cultural objects that open up new frontiers for their diagnosis, monitoring and protection. Any work aimed at the scientific study of art and heritage objects comprises a sequence of steps carefully carried out by different experts who have to work in perfect harmony within an interdisciplinary team. Let us focus on microbiological studies carried out on art objects, as an example. The first step, the most delicate and limiting of the whole process is the sampling (Fig. 1). What may seem so trivial in any other microbiological study, in the case of art objects becomes a real challenge. Restorers and curators do not stop developing increasingly sophisticated and delicate techniques to obtain the biological information contained in art objects without damaging them in any way. These techniques include different processes and tools aimed at obtaining the maximum amount of information with the minimum invasiveness of the objects. As an example, an innovative approach developed not long ago is the technique of micro‐aspiration using small nitrocellulose membranes (de Carvalho *et al*., 2019), which allows to obtain all the biological information accumulated on the surface of objects together with deposited dust, impurities and dirt. The next step is the detailed documentation and cataloguing of each of the samples taken (Pinzari *et al*., 2010) and their distribution to the scientific team for the further investigation, which may include material and microbiological analyses. As mentioned above, the amount of sample obtained from these objects limits the subsequent approaches and therefore, it is necessary from the beginning to plan the workflow of analyses in the most effective way in case samples are to be used again in other assays.

**Fig. 1:**
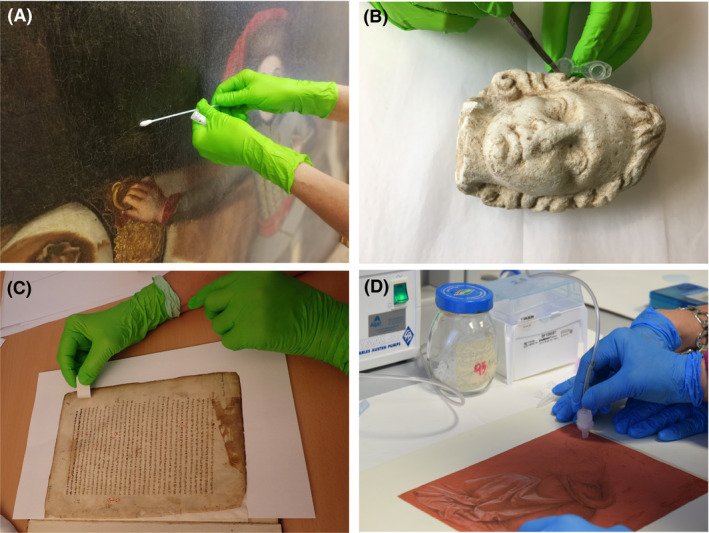
Examples of sampling techniques applied in selected art objects. A. Oil painting on canvas (‘boy holding a dog’ by Johann Georg de Hamilton,18th century, castle Eckartsau, Austria). Sampling was performed using sterile dry cotton swabs by wiping with the swabs across the surface. B. Small female marble head (unknown origin and age, Museum of Art History (KHM), Vienna, Austria). Samples were taken with sterile scalpels by gently scratching from its surface, mainly obtaining powdered material, dust and dirt, without damaging the marble stone. C. Parchment folio (‘Cod. Slav. 136’, 12th century, Austrian National Library, Vienna, Austria). Sampling was done with an eraser by rubbing the surface of the parchment and collecting the fragments detached from it in a sterile test tube. D. Drawing on paper (‘studio di panneggio per una figura inginocchiata’ by Leonardo da Vinci, 15th century, Corsinian Library in Rome, Italy). Sampling was done using the micro‐sampling method, based on a micro‐aspiration with modulable suction force and a filtering apparatus with sterile membranes to trap the particulate material from the surface of the drawing. Credits: pics A, B, C: Piñar and Sterflinger; pic D: ICPAL Rome, Italy.

Concerning material analyses, the study can include the material under investigation itself, but also some micro‐objects, both inorganic and organic, that can be found associated with the surface of the objects, including the dirt and dust accumulated over centuries. These micro‐objects can unravel important information about the manufacture of ancient objects, about their past vicissitudes or about the causes of the deterioration of the materials they contain. The results of critical observation of objects and their microstructures can in some cases be surprising. The careful study of the materials may enable to elucidate certain chemical components attributed to the manufacture of materials or to subsequent restoration works and provide a further tool for the proper diagnosis. These analyses can include scanning electron microscope and energy dispersive X‐ray analysis, UV‐Vis, (Fourier transform) Infrared and Raman spectroscopy, X‐ray and X‐fluorescence as shown in the interesting article by Bicchieri and colleagues (2019).

For the microbiological analyses of art objects, culture‐dependent methods have been widely applied in the past to demonstrate the importance of microorganisms in some deterioration processes. Nevertheless, these methods provide very little information on the true correlation between certain deterioration processes and microorganisms and often yield poor results on the efficiency of restoration treatments. The results obtained might cover only a few organisms that can be cultivated in the laboratory but cannot confirm that the biotic components are responsible of a specific damage, and even if isolation is possible, a single species removed from its natural environment might not necessarily display the same characteristics under laboratory conditions as it does within the material investigated. Therefore, the study of mixed microbial communities colonising a given material is crucial for understanding the various roles played by microorganisms in their degradation.

To this end, molecular techniques have been widely developed in the field of heritage sciences over the last two decades and have provided reliable information about the microbial communities associated with different substrates and materials, enabling sustainable monitoring of restoration treatments. However, they are not free from bias and each step in the workflow influences the results. Starting with the extraction of nucleic acids from art samples, it should be noted that the samples are usually taken in a non‐invasive manner, that is with cotton swabs, membranes or even with erasers or adhesive tapes (Fig. 1). The extraction of DNA/RNA from this type of samples is a very arduous and delicate job that requires a great deal of experience and special care in terms of sterility and scientific accuracy. In addition, the down‐steps are also influencing the results, as the amplification conditions and primer selection used for subsequent PCR‐based methods. Next Generation Sequencing (NGS) technologies have evolved in the last ten years incorporating revolutionary improvements to address the complexities of genomes and metagenomes at an unprecedented speed. NGS enables massive DNA sequencing, providing a much broader view of the real microbial communities that colonise cultural heritage objects. These analyses can be performed using the so‐called ‘shotgun metagenomic approach’, sequencing the entire DNA library representing all the biological information contained in an object. However, this procedure has so far been used sparingly in heritage studies (Teasdale *et al*., 2017; Piñar *et al*., 2020a as examples), partly due to the very low‐quality and low‐concentration of DNA that is often extracted from these samples. The second procedure is based on the sequencing of specific preserved sequences such as ribosomal RNA genes. This approach, called the ‘target amplification approach’, has some advantages, such as reducing the complexity of the data and the possibility of assigning more sequences to specific organisms. The latter approach has been widely used in the field of cultural heritage, as the low‐quality and low‐concentration DNA extracted from these samples can be amplified using degenerated primers and PCR. Many of the studies using this strategy on art and heritage objects have been summarized in the review by Marvasi and colleagues (2019). More recently, third generation sequencing technologies have emerged offering some advantages over the limitations experienced by NGS platforms (Schadt *et al*., 2010). One example is the Nanopore sequencing technology, which involves the use of biological nanopores inserted into a synthetic polymer membrane. Sequencing does not necessarily require an intermediate PCR amplification or chemical labelling step. In addition, this technology offers a pocket‐sized device, the MinION, which offers the advantages of a portable system and the possibility of on‐site analysis. In summary, nanopore sequencing technology, and especially the MinION device, can obviously offer some advantages for practical reasons in cultural heritage studies, such as reduced price, greater simplicity in the workflow and, if necessary, on‐site analysis in museums, archives and repositories. The practical application of this technology in the field of cultural heritage has been launched in the last two years and is still under development, but the few studies that exist so far on various materials, such as wax, stone, textiles and paintings on canvas‐ or paper‐support, have already reported on the advantages of applying this cutting‐edge technology to valuable art objects. Three of these studies, performed on a XVIII Century wax seal (Šoltys *et al*., 2019), the hypogeum of Basilica di San Nicola in Carcere Church in Rom (Grottoli *et al*., 2020), and on some XVII Century funeral textile items (Kisová *et al*., 2020), have reported on the benefits of sequencing long DNA fragments when dealing with ribosomal regions. In other two studies, the Nanopore sequencing technology was applied for the first time together with a whole genome amplification protocol (WGA), either to make a rapid diagnosis of biological infection in canvas paintings of the XVIII and XIX Century (Piñar *et al*., 2020b) or to evaluate the complete microbiomes of some emblematic Leonardo da Vinci’s drawings (Piñar *et al*., 2020c). This latter strategy has made it possible to unify the advantages that this new technology can offer for metagenomic analyses from very low concentrations of DNA and, as it is not based on the amplification of the target regions, to show the real proportions of all the life domains present in the valuable objects under investigation.

Observing this technological development, we can conclude that the strategies used to investigate the microbiological aspects of cultural heritage include a variety of methodologies that cannot be easily standardized and are in permanent development. All methodologies have advantages and disadvantages, and the choice of methods depends in first place on the purpose of the study, but also on the facilities and infrastructure of the Institutions performing the analyses. As scientists, we now have the technology to advance in the field of microbiology and molecular biology applied to cultural heritage. Now, more than ever, we are able to obtain the maximum amount of biological information with the minimum amount of sample from a valuable historical object, something that was unthinkable not long ago. But what does all this technological progress in the field of heritage sciences bring? What can we offer to other professionals such as curators, historians, archaeologists, archivists or philologists who also work in this field?

All these professionals may have different questions aimed at solving different problems, or at providing different approaches to existing information on a particular heritage object. Many of these questions can be answered through the biological information (in the form of DNA) contained on the surface and within the material of the ancient and valuable objects themselves. This biological information, what we call ‘bio‐archive’ or ‘biological pedigree’ is specific to each individual object and can reveal much about the past vicissitudes of the object, its current state of conservation and preservation, and its possible risk of deterioration in the future. But it can also reveal information that can contribute to surprising insights into the objects being investigated, such as the selection of materials at the time of manufacture and the conditions of their storage, or about possible displacements and geographical origins, but also uncover important information about the object's history of use. This information may help to understand many open questions in a variety of fields, as conservation, archaeology, philology, criminology but also, they can contribute to giving an historical benefit to the investigated objects in form of a bio‐archive.

In order for this information to be used as effectively as possible by all professionals working in the heritage sciences, it is of great importance to preserve and transmit to future generations the knowledge, the techniques and the data obtained in an organised and centralised manner for maintenance and access (Sterflinger *et al*., 2018). This implies a challenge, which is the detailed documentation and rigorous storage of all data obtained in the analysis of each valuable object. As an example, those generated by bioinformatic analysis, which form an immense amount of data that has to be stored and at the same time offered with free access for further analysis and comparison with other data obtained, perhaps through other techniques or other objects. This practice would allow the effective transfer of knowledge about valuable objects, but also about the techniques used and developed to reach such knowledge in each professional field involved in the analyses as a whole. Therefore, each sampling and each analysis carried out on a specific art object, as well as the documentation and storage of the results and data obtained in such studies, must be carried out with the best available knowledge and the highest professional responsibility. Finally, our common goal is to save and preserve our cultural heritage, but also the information generated about it, which is an added value to be passed on to future generations.

## Conflict of interest

None declared.
